# A dietary pattern of frequent plant-based foods intake reduced the associated risks for atopic dermatitis exacerbation: Insights from the Singapore/Malaysia cross-sectional genetics epidemiology cohort

**DOI:** 10.1186/s12889-023-16736-y

**Published:** 2023-09-19

**Authors:** Jun Jie Lim, Kavita Reginald, Yee-How Say, Mei Hui Liu, Fook Tim Chew

**Affiliations:** 1 Department of Biological Sciences, Faculty of Science, National University of Singapore, Singapore, 117543 Singapore; 2 Department of Biological Sciences, School of Medicine and Life Sciences, Sunway University, 47500 Petaling Jaya, Selangor, Malaysia; 3 Department of Biomedical Science, Faculty of Science, Universiti Tunku Abdul Rahman (UTAR), 31900 Kampar, Perak, Malaysia; 4 Department of Food Science & Technology, Faculty of Science, National University of Singapore, Singapore, 117543 Singapore

**Keywords:** Atopic dermatitis, Allergic sensitization, Association, Dietary patterns, Ethnic Chinese, Epidemiology, Food types

## Abstract

**Background:**

The prevalence of atopic dermatitis (AD) has been increasing in recent years, especially in Asia. There is growing evidence to suggest the importance of dietary patterns in the development and management of AD. Here, we seek to understand how certain dietary patterns in a Singapore/Malaysia population are associated with various risks of AD development and exacerbation.

**Methods:**

A standardized questionnaire following the International Study of Asthma and Allergies in Childhood (ISAAC) guidelines was investigator-administered to a clinically and epidemiology well-defined allergic cohort of 13,561 young Chinese adults aged 19–22. Information on their sociodemographic, lifestyle, dietary habits, and personal and family medical atopic histories were obtained. Allergic sensitization was assessed by a skin prick test to mite allergens. Spearman’s rank-order correlation was used to assess the correlation between the intake frequencies of 16 food types. Dietary patterns were identified using principal component analysis. Four corresponding dietary scores were derived to examine the association of identified dietary patterns with allergic sensitization and AD exacerbations through a multivariable logistic regression that controlled for age, gender, parental eczema, BMI, and lifestyle factors.

**Results:**

The correlation is the strongest between the intake of butter and margarine (*R* = 0.65). We identified four dietary patterns, “high-calorie foods”, “plant-based foods”, “meat and rice”, and “probiotics, milk and eggs”, and these accounted for 47.4% of the variance in the dietary habits among the subjects. Among these patterns, moderate-to-high intake of “plant-based foods” conferred a negative association for chronic (Adjusted odds ratio (AOR): 0.706; 95% confidence interval (CI): 0.589–0.847; *p* < 0.001) and moderate-to-severe AD (AOR: 0.756; 95% CI: 0.638–0.897; *p* < 0.01). “Meat and rice” and “probiotics, milk and eggs” were not significantly associated with AD exacerbation. While frequent adherence to “high-calorie foods” increased the associated risks for ever AD and moderate-to-severe AD, having a higher adherence to “plant-based foods” diminished the overall associated risks.

**Conclusions:**

Frequent adherence to “plant-based foods” was associated with reduced risks for AD exacerbation in young Chinese adults from Singapore/Malaysia. This provides the initial evidence to support the association between dietary factors and AD. Further research is needed to better understand the pathomechanisms underlying diet and AD exacerbations.

**Supplementary Information:**

The online version contains supplementary material available at 10.1186/s12889-023-16736-y.

## Background

Atopic dermatitis (AD) is a common relapsing inflammatory skin disease that is characterized by pruritus and inflamed eczematous lesions [1]. AD is a growing and significant health concern worldwide, with recent evidence highlighting the association between increased AD prevalence and its impact on the quality of life in Asia [2, 3]. Thus, there is a need for effective prevention and management strategies and further research into the underlying causes of the condition. Overall, AD is a highly heterogenous and complex condition with a multifactorial aetiology that involves epidermal barrier dysfunction, immunologic hypersensitivity, and genetic predisposition [4]. In addition to these factors, there is an increasing interest within the field of allergies to associate dietary habits, patterns, nutrient intakes and AD [5–7]. Given the rising prevalence of AD in Asia, understanding the role of diet in AD development and progression is becoming increasingly important. As AD is commonly recognised as a childhood allergy disease, most studies on AD and diets have only focused on dietary intakes during maternal, prenatal, and postnatal periods [8]. However, young adulthood is also a critical yet overlooked period for establishing long-term dietary habits in preventing AD risk [9]. Furthermore, few studies have evaluated and characterized the specific dietary patterns of AD among adults in Asia. A study using adults from the Korean National Health and Nutrition Examination Survey (KNHANES) highlighted an association between processed foods and meat consumption with increased AD prevalence, but frequent intake of rice reduced the associated odds of AD [10, 11]. Thus, more research is needed to address the lack of regional studies associating diet and AD, especially during young adulthood.

At the population level, dietary pattern analysis is an appropriate and helpful approach to assess the diet-disease relationship by capturing the overall dietary intake and not focusing on individual nutrients [12]. Different foods and beverages are often consumed together in a diet and interactions among them make it challenging to establish their independent effects on health/disease outcomes [13]. Thus, dietary pattern analysis helps provide a more comprehensive understanding of the complex interrelationship between different foods and nutrients as a whole and their association with chronic diseases [14]. Numerous findings have identified dietary patterns associated with a lower risk of cancers, cardiovascular disease, and even inflammatory biomarkers [15–17]. In this study, we seek to i) identify groups of foods that are commonly consumed together and to examine their cumulative effect on AD and ii) gain insight into the complex interplay of multiple dietary factors on the associated risks of AD development and exacerbation. Ultimately, this study provides an important opportunity to advance the understanding of the AD-diet relationship and the findings can inform public health recommendations and interventions aimed at reducing AD risks.

## Methods

### Study design and data collection

This cross-sequential study utilized a well-characterised allergic cohort from the Singapore/Malaysia Cross-sectional Genetics Epidemiology Study (SMCGES). The subjects were composed of mainly university students sampled randomly and consecutively from the National University of Singapore, Universiti Tunku Abdul Rahman, and Sunway University between 2005 and 2022. All participants signed an informed consent form. The study was conducted per the Declaration of Helsinki and Good Clinical Practices. The initial SMCGES cohort was composed of 18,528 subjects. 2432 subjects were subsequently excluded due to missing or invalid data for age, gender, race, and dietary habits. Since a large proportion of Singapore’s population is Chinese (75.2%) [18], 13,561 Chinese were selected for the final analysis to ensure the data was representative of the population and reduced ascertainment bias. A validated questionnaire adopted from the International Study of Asthma and Allergies in Childhood (ISAAC) was investigator-administered to the subjects to obtain information on their socioeconomic, lifestyle and dietary habits, and personal and family atopic medical histories [19].

### Disease definitions

Allergic sensitization was assessed by a skin prick test (SPT) reactivity to common mite allergens from *Blomia tropicalis* and *Dermatophagoides pteronyssinus*. These house dust mites were common yet important allergic sources in the tropics [20]. A subject is SPT positive when a wheal diameter ≥ 3mm appeared in response to either mite allergens when compared to a negative saline control [21]. Histamine was used as a positive control. Those without the presence of a wheal ≥ 3mm for both mite allergens are considered SPT negative. To assess the associated risks for allergic sensitization, a comparison was made between SPT negative subjects as a reference and SPT positive subjects.

Per validated guidelines from the UK Working Party’s diagnostic criteria [22] and Hanifin and Rajka criteria [23], we defined an AD case to be having a recurrent flexural itchy rash and being SPT positive while a non-atopic non-AD control to be SPT negative and not having the recurrent flexural itchy rash. 2316 AD cases were compared to 3650 non-atopic non-AD controls to assess the association between ever AD and dietary patterns among the subjects. In this study, AD exacerbation referred to worsening AD symptoms among subjects with AD. We examined AD exacerbation in terms of chronicity (complete clearance of itchy rash during the last 12 months) and severity (frequency of sleep disturbances at night caused by the itchy rash in the past 12 months). These factors characterised the duration and intensity of the worsening AD symptoms, respectively. Among 2316 AD cases, 809 have chronic AD as their itchy rash failed to clear completely during the last 12 months. While 947 of the AD cases were kept awake at night by their itchy rash in the past 12 months. Non-atopic non-AD controls were compared to chronic AD cases and moderate-to-severe AD cases when assessing for the associated risks for AD chronicity and AD severity, respectively. The distinction between chronic AD and moderate-to-severe AD allows for a more refined analysis of the study outcomes and a better understanding of the specific characteristics that are implicated in subjects with exacerbated AD. A separate analysis was also conducted between those who are AD and SPT positive (N = 2316, 17.1%) vs AD and SPT negative (N = 923, 6.81%) to understand if the presence of an allergic background affects the association between dietary patterns and disease outcome. Please refer to Lim et al. for more information on SPT measurement and disease classification [24].

### Dietary assessment and dietary patterns

A modified section was adopted from ISAAC Phase III Study to examine the dietary habits of 16 food types [25]. The semi-quantitative food-frequency questionnaire (FFQ) was validated for population studies [26]. We asked, “In the past 12 months, how often, on average, did you eat or drink the following: Meat (e.g. Beef, lamb, chicken, pork); Seafood (including fish); Fruits; Vegetables (green and root); Pulses (peas, beans, lentils); Cereals (including bread); Rice; Butter; Margarine; Nuts; Potatoes; Milk; Eggs; Burgers/fast food; Yakult/Vitagen/similar yoghurt drinks (collectively known as probiotic drinks)?” The intake frequencies of 16 food types were recorded as i) never or only occasionally, ii) once or twice per week, and iii) most or all days.

**Fig. 1:**
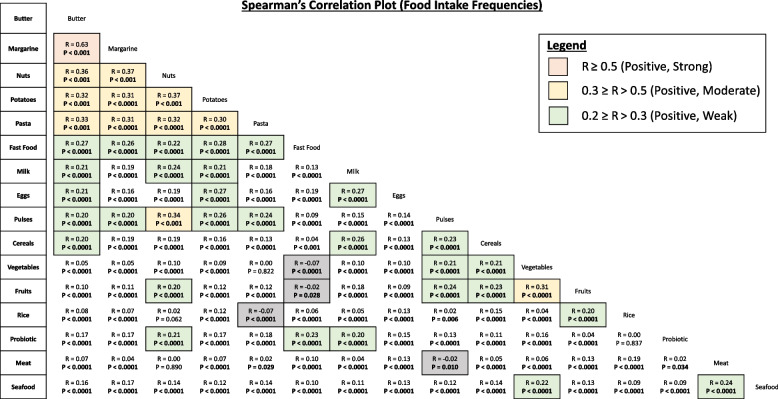
Spearman’s Correlation plot to show 120 specific correlations between 16 food types among 13,561 young Chinese adults in the Singapore/Malaysia Cross-sectional Genetics Epidemiology Study (SMCGES). R represents the Spearman’s rank-order correlation coefficient while p represents the p-value for correlation analysis. Correlations with *R* < 0.20 were negligible. *P* < 0.05 was statistically significant and written in bold

Spearman’s rank-order correlation was conducted to measure the strength and direction of the correlation between food intake frequencies. The Spearman’s correlation coefficient (R) value is from 1 to 0, with *R* ≥ 0.5, 0.3 ≥ *R* > 0.5, and 0.2 ≥ *R* > 0.3 indicating a strong, moderate, and weak correlation, respectively. *R* < 0.2 indicates a negligible correlation between 2 food types [27] (Fig. 1). Following, the Kaiser–Meyer–Olkin (KMO) Measure test and Bartlett's test of sphericity were performed to determine the suitability of data for principal component analysis (PCA). The overall KMO = 0.81 and was considered meritorious, indicating that correlations between food items have excellent sampling adequacy. Bartlett's test of sphericity was also statistically significant (χ2 = 34,153.83, *p* < 0.001), supporting the presence of a sufficiently large variance in the data to justify using PCA.

PCA with orthogonal rotation was used to identify various dietary patterns among the SMCGES cohort. We have chosen the best-representing number of components to retain for further analysis based on the eigenvalues (≥ 1.0) and break point of the scree plot [28, 29] (Supplemental Table 1a, 1b). Among each principal component (PC), those food types with a loading factor ≥ 0.25 were included for the interpretability of the PC (Supplemental Table 1b). The Cronbach’s alpha was analysed in a separate analysis to ensure reliability and consistency in the inclusion and exclusion of food types in each PC (Supplemental Table 1d). Each PC (dietary pattern) was then named based on the food types included. We identified four dietary patterns, PC1 (dietary pattern for high-calorie foods including butter, margarine, nuts, potatoes, and pasta), PC2 (dietary pattern for plant-based foods including vegetables, fruits, and cereals), PC3 (dietary pattern for meat & rice), and PC4 (dietary pattern for probiotics, milk, & eggs). In a separate analysis, the dendrogram obtained from a hierarchical clustering revealed four major clusters which cross-validated and supported the patterns identified by PCA (Supplemental Fig. 2). Apart from the comparison between PC3 and PC4, the other comparisons between various PCs showed to be orthogonal and indicated that the variation captured were independent of each other (Supplemental Fig. 3).

**Fig. 2:**
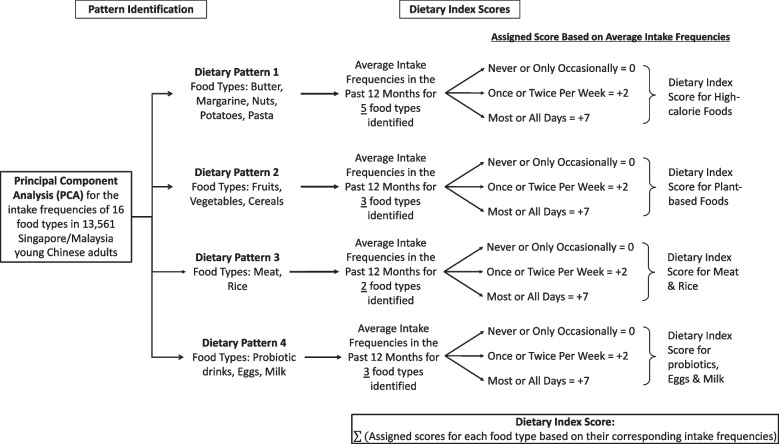
Flowchart illustrating the computation of dietary index score to account for each dietary pattern. These specific dietary index scores were calculated based on the intake frequencies of selected food types among the young Chinese subjects in the Singapore/Malaysia Cross-sectional Genetics Epidemiology Study (SMCGES)

We designed four dietary indices to examine the association between adherence to each dietary pattern and various allergic outcomes. A specific score of + 7 (most or all days), + 2 (once or twice per week), or 0 (never or occasionally) was assigned to the intake frequency of 16 food types following the rubrics by Manousos et al. [30]. Cut-offs for the summation of dietary scores were selected at the 33^rd^ and 66^th^ percentiles based on the preliminary distributional assessment of the SMCGES population. Adequate sensitivity analyses were conducted to ensure reliability and robustness in selecting the cut-offs. Supplemental Table 2 describes the distribution of the range, cut-off scores, and statistical information on the dietary scores while Fig. 2 illustrates the computation of dietary index scores. In the logistic regression analyses for single dietary pattern analysis, the low intake category was used as the reference. For each dietary pattern, subjects who frequently adhered to the specific food types associated with the respective dietary pattern (i.e., high intake of high-calorie foods, high intake of plant-based foods, high intake of meat & rice, and high intake of probiotics, milk & eggs) will have a higher dietary index score. Subsequently, we combined the dietary index of dietary pattern 2 with other dietary indices respectively to assess for any possible interaction between two dietary patterns in nullifying the overall associated risks of allergic outcomes.

### Statistical analysis

All data entries were done using Microsoft Excel (http://office.microsoft.com/en- us/excel/) while subsequent statistical analyses were performed using R program version 2021.09.0.351 (RStudio Team, 2021). Logistic regression was used to model the association between allergic outcomes and various dietary patterns. Potential confounding factors were adjusted in multivariable logistic regression analysis. Results were presented as adjusted odds ratios (AORs) with 95% confidence intervals (CI). The statistical significance of results was defined as *p* < 0.05 and the 95% CI of the AORs not including 1.000. A chi-square analysis was conducted to examine the difference in the distribution of potential confounding variables between disease and non-disease groups. A synergy factor (SF) analysis [31] was conducted to evaluate the interaction between dietary indices in influencing allergic outcomes.

## Results

### Disease prevalence and population demographics

Among 13,561 Chinese from Singapore/Malaysia, most subjects were SPT positive to either mite allergens (*N* = 8840; 65.2%). It was unsurprising as previous allergy studies have supported the high sensitization rate to tropics house dust mites [20, 21]. The ever AD prevalence was high (17.1%) and consistent with the changing natural history of AD disease in Asia [2, 3]. A smaller proportion of AD cases have chronic (5.97%) and moderate-to-severe (6.98%) AD (Table 1). Compared to a hospital setting, where patients were diagnosed with more severe allergic symptoms, the collection conducted in a university setting typically sampled a larger and more diverse population to increase the generalizability of the findings [32].

**Table 1:** Demographic of 13,561 young Chinese adults by allergic disease outcomes in the Singapore/Malaysia Cross-sectional Genetics Epidemiology Study (SMCGES) cohort

	**SPT Negative (*N* = 4622)**	**SPT Positive (*N* = 8840)**	**Chi-square p-value (SPT negative vs. SPT positive)**	**Non-atopic Non-AD Controls (*N *= 3650)**	**AD Cases (*N* = 2316)**	**Chi-square p-value (non-atopic non-AD controls vs. AD cases)**	**Chronic AD Cases (*N* = 809)**	**Chi-square p-value (non-atopic non-AD controls vs. Chronic AD cases)**	**Moderate-to-severe AD Cases (*N* = 947)**	**Chi-square p-value (non-atopic non-AD controls vs. Moderate-to-severe AD cases)**
**Mean Age** ± standard deviation	22.83 ± 6.58	22.35 ± 5.48	-	22.73 ± 6.45	22.41 ± 5.93	-	22.42 ± 6.09	-	22.46 ± 5.89	-
**Gender**										
Male	1308 (28.3%)	4161 (47.1%)	** < 2.200 × 10**^**–16**^	1068 (29.3%)	963 (41.6%)	** < 2.200 × 10**^**–16**^	331 (40.9%)	**1.349 × 10**^**–10**^	370 (39.1%)	**8.268 × 10**^**–9**^
Female	3314 (71.7%)	4679 (52.9%)		2582 (70.7%)	1353 (58.4%)		478 (59.1%)		577 (60.9%)	
**Parental History of Allergic Disease**										
No	3727 (80.6%)	6907 (78.1%)	**5.273 × 10**^**–5**^	3050 (83.6%)	1542(66.6%)	** < 2.200 × 10**^**–16**^	527 (65.1%)	** < 2.200 × 10**^**–16**^	370 (39.1%)	** < 2.200 × 10**^**–16**^
Yes	795 (17.2%)	1785 (20.2%)		518 (14.2%)	741 (32.0%)		271 (33.5%)		577 (60.9%)	
NA	100	148	-	82	33		11		6	
**BMI, Asian Class (kg/m**^**2**^**)**										
Healthy (18.0–23.0)	2590 (56.0%)	4806 (54.4%)	**7.992 × 10**^**–3**^	2060 (56.4%)	1207 (52.1%)	**8.933 × 10**^**–5**^	430 (53.2%)	**7.117 × 10**^**–4**^	479 (50.6%)	**1.624 × 10**^**–4**^
Underweight (< 18.0)	863 (18.7%)	1442 (16.3%)		697 (19.1%)	391 (16.9%)		122 (15.1%)		164 (17.3%)	
Overweight (> 23.0)	687 (14.9%)	1396 (15.8%)		519 (14.2%)	411 (17.7%)		149 (18.4%)		181 (19.1%)	
N/A	482	1196	-	374	307	-	108	-	123	-
**Use of TV/computer (h/day)**										
< 1 h	750 (16.2%)	1462 (16.5%)	**7.487 × 10**^**–4**^	618 (16.9%)	313 (13.5%)	**1.741 × 10**^**–4**^	94 (11.6%)	**4.175 × 10**^**–4**^	115 (12.1%)	**2.278 × 10**^**–5**^
1 to 3 h	1471 (31.8%)	3030 (34.3%)		1191 (32.6%)	720 (31.1%)		274 (33.9%)		279 (29.5%)	
> 3 h to 5 h	1139 (24.6%)	2202 (24.9%)		904 (24.8%)	606 (26.2%)		194 (24.0%)		257 (27.1%)	
> 5 h	1237 (26.8%)	2099 (23.7%)		920 (25.2%)	669 (28.9%)		243 (30.0%)		293 (30.9%)	
NA	25	47	-	17	8	-	4	-	3	-
**Engagement of Physical Activity (times/week)**										
Never or only occasionally	1819 (39.4%)	2796 (31.6%)	** < 2.200 × 10**^**–16**^	1418 (38.8%)	765 (33.0%)	**4.223 × 10**^**–7**^	276 (34.1%)	**9.387 × 10**^**–3**^	329 (34.7%)	**3.733 × 10**^**–3**^
Once or twice per week	2310 (50.0%)	4792 (54.2%)		1841 (50.4%)	1223 (52.8%)		420 (51.9%)		488 (51.5%)	
Most or all days	458 (9.91%)	1190 (13.5%)		365 (10.0%)	311 (13.4%)		104 (12.9%)		126 (13.3%)	
NA	35	62	-	26	17	-	9	-	4	-
**Use of Alcohol**										
Non-drinker	2151 (46.5%)	3651 (41.3%)	**4.575 × 10**^**–6**^	1731 (47.4%)	941 (40.6%)	**9.328 × 10**^**–6**^	346 (42.8%)	**4.657 × 10**^**–2**^	395 (41.7%)	**9.936 × 10**^**–3**^
Occasional	2179 (47.1%)	4439 (50.2%)		1684 (46.1%)	1190 (51.4%)		403 (49.8%)		479 (50.6%)	
Frequent	88 (1.90%)	189 (2.14%)		65 (1.80%)	48 (2.10%)		19 (2.35%)		21 (2.22%)	
NA	204	561	-	170	137	-	41	-	52	-
**Parental Education**										
Primary Education	940 (20.3%)	2047 (23.2%)	** < 2.200 × 10**^**–16**^	775 (21.2%)	505 (21.8%)	**5.995 × 10**^**–3**^	167 (20.6%)	**3.078 × 10**^**–4**^	207 (21.9%)	**2.781 × 10**^**–2**^
Secondary Education	2024 (43.8%)	4273 (48.3%)		1594 (43.7%)	1104 (47.7%)		414 (51.2%)		459 (48.5%)	
Tertiary Education	1436 (31.1%)	2161 (24.4%)		1110 (30.4%)	628 (27.1%)		198 (24.5%)		253 (26.7%)	
NA	222	359	-	171	79	-	30	-	28	-

Abbreviations: *AD* Atopic dermatitis, *BMI* Body mass index, *NA* Not applicable

^1^Allergic sensitization is assessed by a positive skin prick test (SPT) response to either one of the two common house dust mites (*Blomia tropicalis* and *Dermatophagoides pteronyssinus*)

^2^AD is defined to having a positive SPT response and having AD clinical symptoms (recurrent itchy rash that was coming and going for at least 6 months in the flexural areas). For chronic AD subjects, their itchy rash was not cleared completely in the last 12 months, while moderate-to-severe AD subjects experienced sleep disturbances at night in the last 12 months

^3^Parental history of atopic disease is defined by the presence of either paternal and/or maternal medical history of AD or allergic asthma or allergic rhinitis from the immediate family

From the chi-square analysis, there were significant differences in the distribution for all variables between the corresponding disease (SPT positive, AD manifestations) and control (SPT negative, non-AD) groups (Table 1). In general, the mean age in the SMCGES cohort is around aged 22 and comprised of more female subjects. The differences in BMI distribution, engagement in physical activities, and use of alcohol were the most significant between non-atopic non-AD controls and AD cases. Interestingly, the differences in parental education differed most significantly between SPT negative subjects and SPT positive subjects. Finally, a comparison between non-atopic non-AD controls and moderate-to-severe AD showed the most significant difference in the use of TV/computer. As obesity, genetics, socioeconomic, and lifestyle factors have been reported to be well-established risk factors for AD [33–38], we controlled for these potential confounding factors in our multivariable analysis. It ensured that our subsequent analyses better estimated the true effect size and improved causal inference.

### Specific correlation between food intake frequencies

The correlation between butter and margarine was the strongest (*R* = 0.63, *P* < 0.001) among the 120 different correlations identified, with the only correlation coefficient *R* > 0.50 (Fig. 1). There were 11 significant moderate correlations, with the most correlations between food types involving nuts, margarine, potatoes, and pasta. Among those moderately correlated, the correlation coefficient was the highest for the intake frequency of margarine/nuts (*R* = 0.37, *P* < 0.001) and the lowest for potatoes/pasta (*R* = 0.30, *P* < 0.0001). Most correlations were weak, with 29 significant correlations having 0.20 ≥ R^2^ > 0.30. Interestingly, some correlations were negligible, and these included the intake between burgers/fast food and vegetables (*R* = -0.07, *P* < 0.0001), burgers/fast food and fruits (*R* = -0.02, *P* < 0.05), pasta and rice (*R* = -0.07, *P* < 0.0001), and meat and pulses (*R* = -0.02, *P* < 0.05).

### Dietary patterns and association with allergic sensitization, AD, and AD exacerbations

Overall, dietary patterns 1, 2, 3, and 4 explained 22.5%, 10.2%, 8.19%, and 6.68% of the variance in dietary habits, respectively. The cumulative variance explained by the four dietary patterns was 47.4% **(**Supplemental Table 1a). Dietary pattern 1, “high-calorie foods”, was characterized by the intake of high-fat (butter, margarine, and nuts) and high-carbohydrate (potatoes and pasta) foods with high positive loading factors. Dietary pattern 2, “plant-based foods”, was characterized by foods of plant origin, high fibres and vitamins. Another striking aspect was foods found in dietary pattern 1 reflected a negative loading factor in dietary pattern 2 (Supplemental Table 1c). Dietary pattern 3, “meat and rice”, was characterized by foods that formed the key component of an Asian meal, providing primary sources of protein and carbohydrates. Apart from rice, the loading factors of the other three foods (vegetables, fruits, and cereals) in dietary pattern 2 and pulses (another “plant-based” food type) in dietary pattern 3 were negative. Finally, dietary pattern 4, “probiotics, milk, and eggs” was characterized by the intake of probiotic drinks, milk, and eggs. Most importantly, the findings in PCA analysis aligned with those in Spearman’s rank-order correlation and hierarchical clustering.

After establishing four specific dietary patterns among our SMCGES population, we evaluated their associations with various allergic outcomes. In the multivariable model, frequent adherence to “high-calorie foods” was significantly associated with increased risks of allergic sensitization (AOR: 1.208; 95% CI: 1.098–1.330; *p* < 0.001), ever AD (AOR: 1.235; 95% CI: 1.070–1.425; *p* < 0.01), and moderate-to-severe AD (AOR: 1.334; 95% CI: 1.097–1.623; *p* < 0.01) **(**Table 2). Whereas moderate-to-high intake of “plant-based foods” reduced the associated risks for all allergic outcomes. The associated odds for chronic AD were the most pronounced (AOR: 0.706; 95% CI: 0.589–0.847; *p* < 0.001) (Table 2). On the other hand, “meat and rice” and “probiotics, milk, and eggs” dietary patterns were not associated with allergic outcomes. Finally, it was shown in a separate analysis between atopic cases and non-atopic cases that these associations were all insignificant (Supplemental Table 3). These results accentuated that an improvement in dietary patterns was more critical in modulating the associated risks among those with a background of allergic sensitization.

**Table 2:** Association between a) dietary pattern 1 (high-calorie foods) b) dietary pattern 2 (plant-based foods), c) dietary pattern 3 (meat & rice), d) dietary pattern 4 (probiotics, milk, & eggs) and various allergic disease outcomes among 13,561 young Chinese adults from the Singapore/Malaysia Cross-sectional Genetics Epidemiology Study (SMCGES) cohort

Multivariable Logistic Regression												
Dietary Patterns	**Allergic Sentization**^**1**^(SPT Negative [*N* = 4622] vs. SPT Positive [*N* = 8840])			**Ever AD**^**2**^(Non-atopic Non-AD Controls [*N* = 3650] vs. AD Cases [*N* = 2316])			**Chronic AD**^**2**^(Non-atopic Non-AD Controls [*N* = 3650] vs. Chronic AD Cases [*N* = 809])			**Moderate-to-severe AD**^**2**^(Non-atopic Non-AD Controls [*N* = 3650] vs. Moderate-to-severe AD Cases [*N* = 947])		
	AOR	95% CI	*p*	AOR	95% CI	*p*	AOR	95% CI	*p*	AOR	95% CI	*p*
a) **Dietary Pattern 1 (High-calorie Foods)**												
Low Intake of High Calorie Foods (*N* = 4652)	1.000	REF	-	1.000	REF	-	1.000	REF	-	1.000	REF	-
Moderate Intake of High Calorie Foods (*N* = 3677)	1.124	1.011–1.251	**3.000 × 10**^**–2**^	1.241	1.060–1.453	**7.000 × 10**^**–3**^	1.273	1.011–1.603	**4.000 × 10**^**–2**^	1.293	1.041–1.606	**2.000 × 10**^**–2**^
High Intake of High Calorie Foods (*N* = 5232)	1.237	1.121–1.366	**2.28 × 10**^**–5**^	1.243	1.074–1.439	**4.000 × 10**^**–3**^	1.245	1.004–1.545	**4.600 × 10**^**–2**^	1.329	1.089–1.625	**5.000 × 10**^**–3**^
b) **Dietary Pattern 2 (Plant-based Foods)**												
Low Intake of Plant-based Foods (*N* = 4979)	1.000	REF	-	1.000	REF	-	1.000	REF	-	1.000	REF	-
Moderate-to-high Intake of Plant-based Foods (*N* = 8582)	0.917	0.839–1.001	5.330 × 10^–2^	0.819	0.719–0.932	**2.521 × 10**^**–3**^	0.708	0.588–0.854	**2.900 × 10**^**–4**^	0.750	0.630–0.894	**1.000 × 10**^**–3**^
c) **Dietary Pattern 3 (Meat & Rice)**												
Low Intake of Meat & Rice (*N* = 3056)	1.000	REF	-	1.000	REF	-	1.000	REF	-	1.000	REF	-
Moderate-to-high Intake of Meat & Rice (*N* = 10,505)	1.101	0.995–1.218	6.100 × 10^–2^	0.995	0.857–1.158	9.524 × 10^–1^	0.969	0.780–1.210	7.813 × 10^–1^	0.868	0.712–1.061	1.642 × 10^–1^
d) **Dietary Pattern 4 (Probiotics, Milk & Eggs)**												
Low Intake of Probiotics, Milk & Eggs (*N* = 5217)	1.000	REF	-	1.000	REF	-	1.000	REF	-	1.000	REF	-
Moderate Intake of Probiotics, Milk & Eggs (*N* = 2182)	1.045	0.921–1.185	4.972 × 10^–1^	1.017	0.845–1.224	8.554 × 10^–1^	0.902	0.682–1.184	4.611 × 10^–1^	0.970	0.754–1.242	8.101 × 10^–1^
High Intake of Probiotics, Milk & Eggs (*N* = 6162)	0.977	0.891–1.071	6.233 × 10^–1^	0.982	0.857–1.126	7.992 × 10^–1^	0.990	0.912–1.208	9.231 × 10^–1^	0.904	0.752–1.088	2.864 × 10^–1^

Multivariable logistic regression adjusted for age, gender, body mass index, parental history of allergic diseases, sedentary lifestyles, engagement of physical activities, use of alcohol, and parental education. A *p* value < 0.05 from the multivariable logistic is considered statistically significant and is written in bold

^1^Subjects with an allergic sensitization to either one of the two common house dust mites (*Blomia tropicalis* and *Dermatophagoides pteronyssinus*) examined by a skin prick test (SPT). A positive SPT reaction is marked with a wheal diameter of more than 3mm as compared to the saline negative control

^2^AD is defined to be having a positive SPT and having AD clinical symptoms (recurrent itchy rash that was coming and going for at least 6 months in the flexural areas). For chronic AD subjects, their itchy rash was not cleared completely in the last 12 months, while moderate-to-severe AD subjects experienced sleep disturbances at night in the last 12 months. All AD phenotypes were compared to the non-atopic non-AD controls as reference

Although there were concerns about food allergy influencing dietary choices and subsequent pattern identification, the intake frequencies of common food allergens such as nuts, eggs, and milk did not vary significantly between those with and without allergic sensitization (Supplemental Table 4). Thus, this suggested that intentional avoidance and practices of strict diet elimination were unlikely and would not affect the adherence to dietary patterns observed. Moreover, this finding was consistent with the reported low prevalence rates of food allergy in Singapore [39].

### Interactions between dietary patterns in influencing allergic outcomes

Although there was a shift from studying single nutrients and foods towards the impact of overall dietary patterns, most studies often neglected that individuals also consumed a combination of dietary patterns and that the cumulative effects might affect disease risk differently based on the combinations [40]. Thus, we seek to analyze the associations of different combined dietary patterns to increase our understanding of how they affect allergic sensitization and AD exacerbations.

There was strong initial evidence that a “plant-based foods” dietary pattern reduced the associated odds while “high-calorie foods” increased the associated risks for exacerbated AD, respectively. Unsurprisingly, the associated AORs on all allergic outcomes were the lowest for a dietary pattern of low intake of “high-calorie foods” and high intake of “plant-based foods”. Moreover, having a high intake of “plant-based foods” regardless of “high-calorie foods” was sufficient to be negatively associated with moderate-to-severe AD. A dose-dependent reduction in the associated AOR was also observed with increased intake of “plant-based foods” while lowering intake of “high-calorie foods” (Table 3a). While only a dietary pattern of moderate-to-high intake of both “meat & rice” and “plant-based foods” was significantly associated with reduced AORs for chronic AD (AOR: 0.716; 95% CI: 0.535–0.966; *p* < 0.05) (Table 3b). Interestingly, lowering the intake of “probiotics, milk, & eggs” alongside a high intake of “plant-based foods” gradually decreased the associated AORs for chronic AD as well (Table 3c). Finally, a SF analysis was used to understand if the interactions between “plant-based foods” and other selected dietary patterns would exhibit antagonism (lesser than additive effects) in influencing the susceptibility of allergic sensitization and AD exacerbations. It was revealed that only having “plant-based foods” interacted significantly with “high-calorie foods” in reducing the overall associated risks for allergic sensitization and AD chronicity (Supplemental Table 5). Although there were some differences in the stratified ORs for other allergic outcomes (Table 3), this was not reflected in the SF analysis. However, SF was important to understand antagonistic interactions between dietary patterns and it would provide valuable insights into their complex interplay while potentially guides the development of dietary interventions in the future. Taken together, these results suggested that high adherence to a “plant-based foods” diet diminishes the associated risks of high adherence to “high-calorie foods” and possibly other diets on allergic sensitization and AD exacerbations.

**Table 3:** Association between the combined dietary patterns of a) high-calorie foods and plant-based foods, b) meat & rice and plant-based foods, and c) probiotic, milk, & eggs and plant-based foods, and various allergic diseases. Dietary patterns are identified among 13,561 young Chinese adults from the Singapore/Malaysia Cross-sectional Genetics Epidemiology Study (SMCGES) cohort

Multivariable Logistic Regression												
	**Allergic Sentization**^**1**^(SPT Negative [*N* = 4622] vs. SPT Positive [*N* = 8840])			**Ever AD**^**2**^(Non-atopic Non-AD Controls [*N* = 3650] vs. AD Cases [*N* = 2316])			**Chronic AD**^**2**^(Non-atopic Non-AD Controls [*N* = 3650] vs. Chronic AD Cases [*N* = 809])			**Moderate-to-severe AD**^**2**^(Non-atopic Non-AD Controls [*N* = 3650] vs. Moderate-to-severe AD Cases [*N* = 947])		
	AOR	95% CI	*p*	AOR	95% CI	*p*	AOR	95% CI	*p*	AOR	95% CI	*p*
a) **Dietary Pattern 1 (High-calorie Foods) and Dietary Pattern 2 (Plant-based Foods)**												
High Intake of Calorie Foods and Low Intake of Plant-based Foods (*N* = 1439)	1.000	REF	-	1.000	REF	-	1.000	REF	-	1.000	REF	-
High Intake of Calorie Foods and Moderate-to-High Intake of Plant-based Foods (*N* = 3793)	0.957	0.820–1.116	5.754 × 10^–1^	0.819	0.657–1.023	7.805 × 10^–2^	0.743	0.544–1.023	6.553 × 10^–2^	0.691	0.519–0.924	**1.191 × 10**^**–2**^
Moderate Intake of Calorie Foods and Low Intake of Plant-based Foods (*N* = 1301)	0.971	0.802–1.176	7.658 × 10^–1^	0.964	0.734–1.267	7.943 × 10^–1^	0.995	0.679–1.456	9.786 × 10^–1^	0.845	0.591–1.206	3.542 × 10^–1^
Moderate Intake of Calorie Foods and Moderate-to-High Intake of Plant-based Foods (*N* = 2376)	0.837	0.709–0.986	**3.340 × 10**^**–2**^	0.816	0.644–1.034	9.261 × 10^–2^	0.748	0.534–1.051	9.205 × 10^–2^	0.700	0.514–0.955	**2.407 × 10**^**–2**^
Low Intake of Calorie Foods and Low Intake of Plant-based Foods (*N* = 2239)	0.861	0.726–1.021	8.611 × 10^–2^	0.833	0.472–1.065	1.447 × 10^–1^	0.871	0.618–1.232	4.335 × 10^–1^	0.724	0.525–1.000	**4.919 × 10**^**–2**^
Low Intake of Calorie Foods and Moderate-to-High Intake of Plant-based Foods (*N* = 2413)	0.723	0.613–0.852	**1.100 × 10**^**–4**^	0.600	0.472–0.763	**2.990 × 10**^**–5**^	0.495	0.348–0.705	**9.390 × 10**^**–5**^	0.475	0.344–0.655	**5.520 × 10**^**–6**^
b) **Dietary Pattern 3 (Meat & Rice) and Dietary Pattern 2 (Plant-based Foods)**												
Low Intake of Meat & Rice and Low Intake of Plant-based Foods (*N* = 1412)	1.000	REF	-	1.000	REF	-	1.000	REF	-	1.000	REF	-
Low Intake of Meat & Rice and Moderate-to-high Intake of Plant-based Foods (*N* = 1644)	0.879	0.736–1.051	1.571 × 10^–1^	0.953	0.730–1.245	7.236 × 10^–1^	0.694	0.473–1.017	6.058 × 10^–2^	0.943	0.666–1.339	7.400 × 10^–1^
Moderate-to-high Intake of Meat & Rice and Low Intake of Plant-based Foods (*N* = 3567)	1.088	0.928–1.276	2.976 × 10^–1^	1.147	0.905–1.455	2.577 × 10^–1^	1.001	0.725–1.393	9.940 × 10^–1^	1.063	0.780–1.459	7.013 × 10^–1^
Moderate-to-high Intake of Meat & Rice and Moderate-to-high Intake of Plant-based Foods (*N* = 6938)	0.997	0.860–1.154	9.661 × 10^–1^	0.895	0.718–1.118	3.239 × 10^–1^	0.712	0.527–0.972	**2.922 × 10**^**–2**^	0.748	0.560–1.008	5.265 × 10^–2^
c) **Dietary Pattern 4 (Probiotics, Milk, & Eggs) and Dietary Pattern 2 (Plant-based Foods)**												
Low Intake of Probiotics, Milk, & Eggs, and Low Intake of Plant-based Foods (*N* = 2517)	1.000	REF	-	1.000	REF	-	1.000	REF	-	1.000	REF	-
Low Intake of Probiotics, Milk, & Eggs, and Moderate-to-high Intake of Plant-based Foods (*N* = 2700)	1.017	0.886–1.168	8.059 × 10^–1^	0.945	0.770–1.161	5.914 × 10^–1^	0.674	0.499–0.910	**1.022 × 10**^**–2**^	0.931	0.707–1.226	6.095 × 10^–1^
Moderate Intake of Probiotics, Milk, & Eggs, and Low Intake of Plant-based Foods (*N* = 847)	1.053	0.862–1.290	6.153 × 10^–1^	1.181	0.887–1.570	2.539 × 10^–1^	0.860	0.561–1.294	4.766 × 10^–1^	1.203	0.823–1.744	3.345 × 10^–1^
Moderate Probiotics, Milk, & Eggs, and Moderate-to-high Intake of Plant-based Foods (*N* = 1335)	1.055	0.894–1.247	5.255 × 10^–1^	0.878	0.683–1.127	3.080 × 10^–1^	0.678	0.469–0.971	**3.646 × 10**^**–2**^	0.786	0.556–1.103	1.674 × 10^–1^
High Intake of Probiotics, Milk, & Eggs, and Low Intake of Plant-based Foods (*N* = 1615)	1.171	0.998–1.373	5.270 × 10^–2^	1.206	0.957–1.521	1.122 × 10^–1^	1.086	0.787–1.494	6.149 × 10^–1^	1.198	0.880–1.627	2.486 × 10^–1^
High Probiotics, Milk, & Eggs, and Moderate-to-high Intake of Plant-based Foods (*N* = 4547)	0.933	0.824–1.055	2.685 × 10^–1^	0.881	0.734–1.058	1.739 × 10^–1^	0.738	0.572–0.956	**2.043 × 10**^**–2**^	0.779	0.609–0.999	**4.794 × 10**^**–2**^

Results were obtained from a multivariable logistic regression adjusted for age, gender, parental history of allergic disease, BMI, use of alcohol, use of TV/computer, engagement of physical activities, and parental education. *P* value < 0.05 from the multivariable logistic regression is considered statistically significant and bolded

^1^Subjects with allergic sensitization is defined by a positive skin prick test (SPT) response to common house dust mites (*Blomia tropicalis* and *Dermatophagoides pteronyssinus*) with a wheal diameter of more than 3mm as compared to the saline negative control. Allergic sensitization was compared between SPT negative subjects (as reference) and SPT positive subjects

^2^AD is defined to be having a positive SPT response and having AD clinical symptoms (recurrent itchy rash that was coming and going for at least 6 months in the flexural areas). For chronic AD subjects, their itchy rash was not cleared completely in the last 12 months, while moderate-to-severe AD subjects experienced sleep disturbances at night in the last 12 months. All AD phenotypes were compared to the non-atopic non-AD controls as reference

## Discussion

Characterization of dietary patterns and their association with AD is important for our increased understanding of diets as key modifiable risk factors. To the best of our knowledge, this is the first study in Singapore/Malaysia to evaluate the association between dietary patterns, allergic sensitization, and AD exacerbations. Among a large cohort of clinically and epidemiologically well-defined individuals with AD, we observed strong correlations between food intake frequencies and identified four unique empirically-defined dietary patterns. Of these, the dietary pattern of “high-calorie foods” was positively associated with allergic sensitization and AD exacerbations. A dietary pattern of “plant-based foods”, however, significantly reduced the associated risks. Frequent adherence to “plant-based foods” dietary patterns to other dietary patterns (“high-calorie foods”, “meat & rice”, and “probiotics, milk & eggs”) further attenuated the associated risks of chronic AD.

Diet remained a critical component of health promotion and disease prevention strategies, and early adulthood was suggested to be a significant period where dietary habits can be modified [41]. A study investigating patients’ perceptions of the role of diet in AD revealed a lack of proper dietary modification and nutritional counselling among AD patients despite them recognizing the importance of diet as a management tool in AD [42]. The dietary patterns identified in our study resembled dietary patterns previously associated with allergy and AD risks [25, 43–46]. These dietary patterns comprised energy-dense foods such as fast foods, potatoes, butter, and margarine. Typically, energy-dense foods are high in calories, saturated and trans fats, added sugar, and sodium which altogether can promote oxidative stress and inflammation to potentially affect allergic sensitization and AD [47–49]. Our findings are also supportive of the dietary habits in Singapore which excessive total fats, sodium, and sugar intake constituted a major concern [50]. We have also highlighted that lowering the intake of “high-calorie foods” can significantly reduce the associated risks of allergic outcomes among young Chinese adults in Singapore. These findings are of great interest due to their implications in nutrition education among young adults to help them make more informed personal food choices.

The consumption of fruits and vegetables has been extensively associated with a reduction in inflammatory biomarkers such as TNF-alpha and C-reactive protein, and improved AD symptoms [51, 52]. Some studies showed that adherence to a vegetarian diet downregulated monocytes prostaglandin E2 and peripheral eosinophils in those with severe AD [53]. Our results add to the understanding that higher adherence to “plant-based foods” than “high-calorie foods” reduced the associated odds for allergic sensitization and exacerbated AD conditions. Thus, we postulated that an increased intake of these plant-based foods in diets provided sufficient phytochemicals (flavonoids, carotenoids etc.) and anti-oxidant nutrients (vitamin C and vitamin E etc.) to regulate various immune cells and their secreted inflammatory cytokines to overcome the pro-inflammatory activity imposed by the consumption of high-calorie/ meat and fast food [54]. Additional prospective research is needed to confirm these findings. A well-designed randomized controlled trial involving feeding AD patients a diet rich in nutrient-dense, plant-based foods is recommended.

Although some studies attribute milk, dairy products, and probiotics to be largely anti-inflammatory in non-allergic individuals, the overall literature remained controversial and inconclusive [55, 56]. A trial conducted on young children suggested the avoidance of eggs and cows’ milk to improve AD severity [57]. It may be more useful to avoid eggs and dairy products during early life among those with food allergies. It is also important to note that the effects of probiotics on AD differ individually. In addition, commercial probiotic products varied widely in terms of the number, type, and function of commercial strains which may cause varying changes to the composition, diversity, and even function of the gut microbiome among individuals. Thus, it may partly explain why we did not find any association between a dietary pattern of “probiotics, milk, and eggs” and allergic outcomes in our study of young Chinese adults.

Due to the cross-sectional nature of this study, we cannot necessarily show that dietary patterns of “plant-based foods” protect against AD exacerbation. Furthermore, the classification and labelling of dietary patterns were based on subjective judgment and may differ among some individuals. However, we have performed sensitivity analyses to ensure the robustness of the results to different numbers of principal components and food types alongside their associations with allergic outcomes. Additionally, the use of a standardized FFQ adopted from ISAAC ensured that dietary data are collected in a consistent, reproducible manner that is comparable across studies. We have also minimized recall and measurement bias by providing direct assistance and clarification to subjects during the collection in ensuring completeness and accuracy in the dietary data. In the future, we will conduct an inter-comparison in another large and well-characterised independent cohort to confirm the validity of the identified dietary patterns and associations with AD exacerbations.

## Conclusions

To conclude, we have derived four specific dietary patterns among our SMCGES cohort and found that a higher adherence to “plant-based foods” than “high-calorie foods” was negatively associated with allergic sensitization, chronic, and moderate-to-severe AD among young Chinese adults. As most people eat and drink a myriad of foods, it is easier to implement dietary intervention based on dietary patterns rather than on a single food type. Individuals with an allergic background and/or AD need to discuss with their clinicians and nutritionists their dietary choices while considering their unique needs.

## Fundings

F.T.C. has received research support from the National University of Singapore, Singapore Ministry of Education Academic Research Fund, Singapore Immunology Network (SIgN), National Medical Research Council (NMRC) (Singapore), Biomedical Research Council (BMRC) (Singapore), National Research Foundation (NRF) (Singapore), Singapore Food Agency (SFA), and the Agency for Science Technology and Research (A*STAR) (Singapore); Grant Numbers are N-154–000-038–001, R-154–000-191–112, R-154–000-404–112, R-154–000-553–112, R-154–000-565–112, R-154–000-630–112, R-154–000-A08-592, R-154–000-A27-597, R-154–000-A91-592, R-154–000-A95-592, R-154–000-B99-114, SIgN-06–006, SIgN-08–020, NMRC/1150/2008, OFIRG20nov-0033, BMRC/01/1/21/18/077, BMRC/04/1/21/19/315, BMRC/APG2013/108, NRF-MP-2020–0004, SFS_RND_SUFP_001_04, W22W3D0006, H17/01/a0/008 and APG2013/108. F.T.C. has received consulting fees from Sime Darby Technology Centre, First Resources Ltd, Genting Plantation, Olam International and Syngenta Crop Protection, outside the submitted work. All funding agencies had no role in the study design, data collection and analysis, decision to publish, or preparation of the manuscript.

### Supplementary Information

**Additional file 1: Supplemental Fig 1.**

**Additional file 2: Supplemental Fig 2.**

**Additional file 3: Supplemental Fig 3.**

**Additional file 4: Supplemental Table 1.**

**Additional file 5: Supplemental Table 2.**

**Additional file 6: Supplemental Table 3.**

**Additional file 7: Supplemental Table 4. **

**Additional file 8: Supplemental Table 5.**

## Data Availability

The data underlying this article will be shared on reasonable request to the corresponding author (F.T.C.).

## Abbreviations

AD: Atopic Dermatitis
AOR: Adjusted Odds Ratio
BMI: Body Mass Index
CI: Confidence Intervals
HPB: Health Promotion Board
ISAAC: International Study of Asthma and Allergies in Childhood
PCA: Principal Component Analysis
SMCGES: Singapore/Malaysia Cross-sectional Genetics Epidemiology study
SPT: Skin Prick Test
