# Supplementary material for: A dietary pattern of frequent plant-based foods intake reduced the associated risks for atopic dermatitis exacerbation: Insights from the Singapore/Malaysia cross-sectional genetics epidemiology cohort
Source: BMC Public Health. 2023 Sep 19;23:1818. doi: 10.1186/s12889-023-16736-y (PMC10508008; doi:10.1186/s12889-023-16736-y)
Supplement: Supplementary file 1 — Additional file 1: Supplemental Fig 1. [file 12889_2023_16736_MOESM1_ESM.docx]

**Supplemental Figure 1**. Scree plot showing the variation of each principal component from a principal component analysis (PCA) for the intake frequencies of 16 food types among 13,561 young Chinese adults from the Singapore/Malaysia Cross-sectional Genetics Epidemiology Study (SMCGES).
