# Supplementary material for: A dietary pattern of frequent plant-based foods intake reduced the associated risks for atopic dermatitis exacerbation: Insights from the Singapore/Malaysia cross-sectional genetics epidemiology cohort
Source: BMC Public Health. 2023 Sep 19;23:1818. doi: 10.1186/s12889-023-16736-y (PMC10508008; doi:10.1186/s12889-023-16736-y)
Supplement: Supplementary file 4 — Additional file 4: Supplemental Table 1. [file 12889_2023_16736_MOESM4_ESM.docx]

**Supplemental Table 1.** Principal component analysis (PCA) on 16 food types among 13,561 young Chinese adults in the Singapore/Malaysia Cross-sectional Genetics Epidemiology Study (SMCGES) cohort. (**a**) eigenvalues obtained from PCA, alongside with the cumulative variance explained in each principal component (PC), (**b**) loading factors for each selected PC and (**c**) Cronbach’s alpha analysis to determine the reliability in retaining food types under each PC.

| 1. **Eigenvalues and Variance Explained by Each Principal Component** | | | |
| --- | --- | --- | --- |
|  | **Eigenvalues** | **Variance Explained (%)** | **Cumulative Variance Explained (%)** |
| Principal Component 1 (PC1) | 3.5853 | 22.5 | 22.46 |
| Principal Component 2 (PC2) | 1.6179 | 10.2 | 32.6 |
| Principal Component 3 (PC3) | 1.3063 | 8.2 | 40.8 |
| Principal Component 4 (PC4) | 1.0647 | 6.7 | 47.4 |
| Principal Component 5 (PC5) | 1.0220 | 6.4 | 53.8 |
| Principal Component 6 (PC6) | 0.9198 | 5.7 | 59.6 |
| Principal Component 7 (PC7) | 0.8570 | 5.4 | 64.9 |
| Principal Component 8 (PC8) | 0.7641 | 4.7 | 69.7 |
| Principal Component 9 (PC9) | 0.7072 | 4.5 | 74.1 |
| Principal Component 10 (PC10) | 0.6828 | 4.3 | 78.4 |
| Principal Component 11 (PC11) | 0.6787 | 4.2 | 82.6 |
| Principal Component 12 (PC12) | 0.6380 | 4.0 | 86.6 |
| Principal Component 13 (PC13) | 0.6305 | 4.0 | 90.6 |
| Principal Component 14 (PC14) | 0.5962 | 3.7 | 94.3 |
| Principal Component 15 (PC15) | 0.5640 | 3.5 | 97.8 |
| Principal Component 16 (PC16) | 0.3655 | 2.3 | 100.0 |

| 1. **Loading Factors** | | | | |
| --- | --- | --- | --- | --- |
|  | **Principal Component (PC) 1** | **Principal Component (PC) 2** | **Principal Component (PC) 3** | **Principal Component (PC) 4** |
| Butter | **0.3512** | -0.1998 | 0.1044 | -0.3730 |
| Margarine | **0.3419** | -0.2048 | 0.0673 | -0.4137 |
| Nuts | **0.3428** | -0.0998 | -0.1873 | -0.0690 |
| Burgers/fast food | 0.2375 | -0.2957 | 0.2870 | 0.1932 |
| Eggs | 0.2376 | 0.0533 | 0.2233 | **0.4164** |
| Meat (E.g., Beef, lamb, chicken, pork) | 0.0856 | 0.2563 | **0.5656** | 0.0131 |
| Milk | 0.2531 | 0.0786 | -0.0790 | **0.4211** |
| Pasta | **0.2900** | -0.2366 | -0.0938 | -0.0165 |
| Seafood (including fish) | 0.1908 | 0.2325 | 0.2316 | -0.0862 |
| Cereals (including bread) | 0.2249 | **0.2783** | -0.1517 | -0.0548 |
| Rice | 0.0905 | 0.3100 | **0.4221** | -0.1452 |
| Potatoes | **0.3271** | -0.1325 | 0.0351 | 0.0457 |
| Pulses (peas, beans, lentils) | 0.2625 | 0.1331 | -0.3581 | -0.0904 |
| Vegetables (green and root) | 0.1363 | **0.5187** | -0.1024 | -0.1069 |
| Fruits | 0.1988 | **0.3902** | -0.2905 | 0.0504 |
| Probiotic Drinks | 0.2112 | -0.0761 | -0.0563 | **0.4906** |

Loading factors written in bold were included for the interpretability of the PC.

| **ci) Principal Component 1 (PC1)** | | | | |
| --- | --- | --- | --- | --- |
|  | **Test 1** | **Test 2** | **Test 3** | **Test 4** |
| Butter | 0.67 | 0.67 | 0.63 | 0.54 |
| Nuts | 0.67 | 0.70 | 0.69 | 0.77 |
| Margarine | 0.68 | 0.67 | 0.63 | 0.53 |
| Potatoes | 0.68 | 0.72 | 0.72 | - |
| Pasta | 0.69 | 0.73 | - | - |
| Pulses (peas, beans, lentils) | 0.72 | - | - | - |
| Milk | 0.74 | - | - | - |
| Overall Cronbach’s alpha | 0.72 | **0.74** | 0.73 | 0.72 |

Food types with a loading factor ≥ 0.25 were included in the Cronbach’s alpha analysis.

| **cii) Principal Component 2 (PC2)** | | | | |
| --- | --- | --- | --- | --- |
|  | **Test 1** | **Test 2** | **Test 3** | **Test 4** |
| Vegetables (green and root) | 0.34 | 0.33 | 0.37 | 0.24 |
| Fruits | 0.40 | 0.38 | 0.32 | 0.40 |
| Rice | 0.44 | 0.48 | 0.47 | - |
| Cereals (including bread) | 0.41 | 0.40 | - | - |
| Meat | 0.47 | - | - | - |
| Overall Cronbach’s alpha | 0.47 | 0.47 | **0.48** | 0.47 |

Food types with a loading factor ≥ 0.25 were included in the Cronbach’s alpha analysis.

| **ciii) Principal Component 3 (PC3)** | | |
| --- | --- | --- |
|  | **Test 1** | **Test 2** |
| Meat | 0.11 | 0.20 |
| Rice | 0.18 | 0.19 |
| Burgers/fast food | 0.32 | - |
| Overall Cronbach’s alpha | 0.29 | **0.32** |

Food types with a loading factor ≥ 0.25 were included in the Cronbach’s alpha analysis.

**civ) Principal Component 4 (PC4)**

|  | **Test 1** | **Test 2** |
| --- | --- | --- |
| Probiotic Drinks | 0.42 | 0.43 |
| Milk | 0.27 | 0.22 |
| Eggs | 0.33 | - |
| Overall Cronbach’s alpha | **0.44** | 0.33 |

Food types with a loading factor ≥ 0.25 were included in the Cronbach’s alpha analysis.
