# Supplementary material for: A dietary pattern of frequent plant-based foods intake reduced the associated risks for atopic dermatitis exacerbation: Insights from the Singapore/Malaysia cross-sectional genetics epidemiology cohort
Source: BMC Public Health. 2023 Sep 19;23:1818. doi: 10.1186/s12889-023-16736-y (PMC10508008; doi:10.1186/s12889-023-16736-y)
Supplement: Supplementary file 5 — Additional file 5: Supplemental Table 2. [file 12889_2023_16736_MOESM5_ESM.docx]

**Supplemental Table 2.** Information on the dietary score for each selected dietary pattern among 13,561 young Chinese adults.

|  | **Dietary Pattern 1**  **(High Calorie Foods)** | **Dietary Pattern 2**  **(Plant-based Foods)** | **Dietary Pattern 3**  **(Meat & Rice)** | **Dietary Pattern 4**  **(Probiotics, Milk, & Eggs)** |
| --- | --- | --- | --- | --- |
| Food Types Included | Butter, Margarine, Nuts, Potatoes, Pasta | Vegetables, Fruits, Cereals | Meat and Rice | Probiotic Drinks, Milk, Eggs |
| - Score Range | | | | |
| Minimum Dietary Score | 0.00 | 0.00 | 0.00 | 0.00 |
| Maximum Dietary Score | 35.0 | 21.0 | 14.0 | 21.0 |
| 1^st^ Quantile | 4.00 | 11.0 | 14.0 | 6.00 |
| 3^rd^ Quantile | 11.0 | 21.0 | 14.0 | 14.0 |
| Mean | 8.77 | 15.2 | 12.6 | 9.76 |
| Median | 8.00 | 16.0 | 14.0 | 9.00 |
| - Cut-offs | | | | |
| Low Dietary Score^1^ (33^rd^ cut-offs) | ≤4.00 | ≤14.0 | <14.0 | ≤7.00 |
| Moderate Dietary Score^1^ | 5.00-9.00 | 15.0* | ≥15.00 | 8.00-10.0 |
| High Dietary Score^1^ (66^th^ cut-offs) | ≥10.0 | ≥16.0 |  | ≥11.0 |

^1^ Subjects having a lower dietary score for a dietary pattern is indicative of a corresponding low adherence.

*Indicates no subject having the particular dietary score
