# Supplementary material for: A dietary pattern of frequent plant-based foods intake reduced the associated risks for atopic dermatitis exacerbation: Insights from the Singapore/Malaysia cross-sectional genetics epidemiology cohort
Source: BMC Public Health. 2023 Sep 19;23:1818. doi: 10.1186/s12889-023-16736-y (PMC10508008; doi:10.1186/s12889-023-16736-y)
Supplement: Supplementary file 6 — Additional file 6: Supplemental Table 3. [file 12889_2023_16736_MOESM6_ESM.docx]

**Supplemental Table 3.** Association analysis was conducted between non-atopic cases and atopic cases for a) dietary pattern 1 (high-calorie foods) b) dietary pattern 2 (plant-based foods), c) dietary pattern 3 (meat & rice), d) dietary pattern 4 (probiotics, milk, & eggs) among 13,561 young Chinese adults from the Singapore/Malaysia Cross-sectional Genetics Epidemiology Study (SMCGES) cohort.

| **Non-atopic Cases [N = 923] vs. Atopic Cases [N = 2316]** | | | |
| --- | --- | --- | --- |
| 1. **Dietary Pattern 1 (High-calorie Foods)** | | | |
|  | OR | 95% CI | p |
| Low Intake of High Calorie Foods (N = 4652) | 1.000 | REF | - |
| Moderate Intake of High Calorie Foods (N = 3677) | 1.023 | 0.844-1.241 | 8.135 x 10^-1^ |
| High Intake of High Calorie Foods (N =5232) | 1.225 | 1.021-1.469 | **2.910 x 10^-2^** |
|  | AOR | 95% CI | p |
| Low Intake of High Calorie Foods (N = 4652) | 1.000 | REF | - |
| Moderate Intake of High Calorie Foods (N = 3677) | 0.992 | 0.793-1.243 | 9.469 x 10^-1^ |
| High Intake of High Calorie Foods (N =5232) | 1.062 | 0.859-1.313 | 5.759 x 10^-1^ |
| 1. **Dietary Pattern 2 (Plant-based Foods)** | | | |
|  | OR | 95% CI | p |
| Low Intake of Plant-based Foods (N = 4979) | 1.000 | REF | - |
| Moderate-to-high Intake of Plant-based Foods (N = 8582) | 0.952 | 0.813-1.113 | 5.350 x 10^-1^ |
|  | AOR | 95% CI | p |
| Low Intake of Plant-based Foods (N = 4979) | 1.000 | REF | - |
| Moderate-to-high Intake of Plant-based Foods (N = 8582) | 0.943 | 0.783-1.134 | 5.335 x 10^-1^ |
| 1. **Dietary Pattern 3 (Meat & Rice)** | | | |
|  | OR | 95% CI | p |
| Low Intake of Meat & Rice (N = 3056) | 1.000 | REF | - |
| Moderate-to-high Intake of Meat & Rice (N = 10505) | 1.355 | 1.140-1.608 | **5.370 x 10^-4^** |
|  | AOR | 95% CI | p |
| Low Intake of Meat & Rice (N = 3056) | 1.000 | REF | - |
| Moderate-to-high Intake of Meat & Rice (N = 10505) | 1.213 | 0.988-1.486 | 6.382 x 10^-2^ |
| 1. **Dietary Pattern 4 (Probiotics, Milk & Eggs)** | | | |
|  | OR | 95% CI | p |
| Low Intake of Probiotics, Milk & Eggs (N = 5217) | 1.000 | REF | - |
| Moderate Intake of Probiotics, Milk & Eggs (N = 2182) | 1.064 | 0.847-1.341 | 5.970 x 10^-1^ |
| High Intake of Probiotics, Milk & Eggs (N = 6162) | 0.994 | 0.841-1.175 | 9.470 x 10^-1^ |
|  | AOR | 95% CI | p |
| Low Intake of Probiotics, Milk & Eggs (N = 5217) | 1.000 | REF | - |
| Moderate Intake of Probiotics, Milk & Eggs (N = 2182) | 1.050 | 0.802-1.381 | 7.249 x 10^-1^ |
| High Intake of Probiotics, Milk & Eggs (N = 6162) | 0.944 | 0.777-1.146 | 5.596 x 10^-1^ |
