# Supplementary material for: A dietary pattern of frequent plant-based foods intake reduced the associated risks for atopic dermatitis exacerbation: Insights from the Singapore/Malaysia cross-sectional genetics epidemiology cohort
Source: BMC Public Health. 2023 Sep 19;23:1818. doi: 10.1186/s12889-023-16736-y (PMC10508008; doi:10.1186/s12889-023-16736-y)
Supplement: Supplementary file 7 — Additional file 7: Supplemental Table 4. [file 12889_2023_16736_MOESM7_ESM.docx]

**Supplemental Table 4.** Distribution of intake frequencies for selected food types between those with skin prick test (SPT) positive and SPT negative.

| Food Types (Common Food Allergens) | SPT negative subjects  (N = 4622) | SPT positive subjects  (N = 8840) | Chi-square p-value |
| --- | --- | --- | --- |
| **Milk** |  |  |  |
| - Never or Only Occasionally | 1013 (21.9%) | 1900 (14.0%) | 3.211 x 10^-1^ |
| - Once or Twice Per Week/ Most or All Days | 3584 (77.5%) | 6892 (78.0%) |  |
| NA | 25 | 48 | - |
| **Eggs** | | | |
| - Never or Only Occasionally | 156 (3.38%) | 281 (3.18%) | 3.706 x 10^-1^ |
| - Once or Twice Per Week/ Most or All Days | 4436 (96.0%) | 8500 (96.2%) |  |
| NA | 30 | 59 | - |
| **Nuts** | | | |
| - Never or Only Occasionally | 2027(43.9 %) | 3862 (43.7%) | 8.531 x 10^-1^ |
| - Once or Twice Per Week/ Most or All Days | 2569 (55.6%) | 4928 (55.7%) |  |
| NA | 26 | 50 | - |
