# Supplementary material for: A dietary pattern of frequent plant-based foods intake reduced the associated risks for atopic dermatitis exacerbation: Insights from the Singapore/Malaysia cross-sectional genetics epidemiology cohort
Source: BMC Public Health. 2023 Sep 19;23:1818. doi: 10.1186/s12889-023-16736-y (PMC10508008; doi:10.1186/s12889-023-16736-y)
Supplement: Supplementary file 8 — Additional file 8: Supplemental Table 5. [file 12889_2023_16736_MOESM8_ESM.docx]

**Supplemental Table 5.** Synergy factor (SF) analysis to determine the interaction between dietary patterns in influencing allergic outcomes among 13,561 young Chinese adults from the Singapore/Malaysia Cross-sectional Genetics Epidemiology Study (SMCGES) cohort.

| 1. **Dietary Pattern 1 (High-calorie Foods) and Dietary Pattern 2 (Plant-based Foods)** | | | | | |
| --- | --- | --- | --- | --- | --- |
| 1. **Allergic Sensitization (SPT negative subjects vs. SPT positive subjects)** | | | | | |
| Dietary Pattern 1  (High-calorie Foods) | Dietary Pattern 2  (Plant-based Foods) | SPT Negative | SPT Positive | SF P Value | SF Value  (95% CI) |
| High | Low | 436 | 994 | 0.049 | **0.836**  (0.700-0.999) |
| High | Moderate-to-high | 1218 | 2553 |  |  |
| Low | Low | 756 | 1458 |  |  |
| Low | Moderate-to-high | 963 | 1428 |  |  |
| 1. **Ever AD (Non-atopic non-AD controls vs. AD cases)** | | | | | |
| Dietary Pattern 1  (High-calorie Foods) | Dietary Pattern 2  (Plant-based Foods) | Non-atopic Non-AD Controls | AD Cases | SF P Value | SF Value  (95% CI) |
| High | Low | 340 | 286 | 0.242 | 0.857  (0.661-1.110) |
| High | Moderate-to-high | 971 | 644 |  |  |
| Low | Low | 577 | 379 |  |  |
| Low | Moderate-to-high | 800 | 355 |  |  |
| 1. **AD Chronicity (Non-atopic non-AD controls vs. Chronic AD cases)** | | | | | |
| Dietary Pattern 1  (High-calorie Foods) | Dietary Pattern 2  (Plant-based Foods) | Non-atopic Non-AD Controls | Chronic AD Cases | SF P Value | SF Value  (95% CI) |
| High | Low | 340 | 100 | 0.035 | **0.665**  (0.455-0.972) |
| High | Moderate-to-high | 971 | 221 |  |  |
| Low | Low | 577 | 150 |  |  |
| Low | Moderate-to-high | 800 | 107 |  |  |
| 1. **AD Severity (Non-atopic non-AD controls vs. Moderate-to-severe AD cases)** | | | | | |
| Dietary Pattern 1  (High-calorie Foods) | Dietary Pattern 2  (Plant-based Foods) | Non-atopic Non-AD Controls | Moderate-to-severe AD Cases | SF P Value | SF Value  (95% CI) |
| High | Low | 340 | 129 | 0.443 | 0.871  (0.612-1.240) |
| High | Moderate-to-high | 971 | 269 |  |  |
| Low | Low | 577 | 152 |  |  |
| Low | Moderate-to-high | 800 | 134 |  |  |
| 1. **Dietary Pattern 3 (Meat & Rice) and Dietary Pattern 2 (Plant-based Foods)** | | | | | |
| 1. **Allergic Sensitization (SPT negative subjects vs. SPT positive subjects)** | | | | | |
| Dietary Pattern 3  (Meat & Rice) | Dietary Pattern 2  (Plant-based Foods) | SPT Negative | SPT Positive | SF P Value | SF Value  (95% CI) |
| Low | Low | 495 | 898 | 0.664 | 1.039  (0.875-1.233) |
| Low | Moderate-to-high | 656 | 979 |  |  |
| Moderate-to-high | Low | 1098 | 2446 |  |  |
| Moderate-to-high | Moderate-to-high | 2373 | 4517 |  |  |
| 1. **Ever AD (Non-atopic non-AD controls vs. AD cases)** | | | | | |
| Dietary Pattern 3  (Meat & Rice) | Dietary Pattern 2  (Plant-based Foods) | Non-atopic Non-AD Controls | AD Cases | SF P Value | SF Value  (95% CI) |
| Low | Low | 362 | 243 | 0.279 | 0.871  (0.678-1.119) |
| Low | Moderate-to-high | 504 | 292 |  |  |
| Moderate-to-high | Low | 861 | 665 |  |  |
| Moderate-to-high | Moderate-to-high | 1923 | 1116 |  |  |
| 1. **AD Chronicity (Non-atopic non-AD controls vs. Chronic AD cases)** | | | | | |
| Dietary Pattern 3  (Meat & Rice) | Dietary Pattern 2  (Plant-based Foods) | Non-atopic Non-AD Controls | Chronic AD Cases | SF P Value | SF Value  (95% CI) |
| Low | Low | 362 | 97 | 0.741 | 0.941  (0.656-1.350) |
| Low | Moderate-to-high | 504 | 96 |  |  |
| Moderate-to-high | Low | 861 | 247 |  |  |
| Moderate-to-high | Moderate-to-high | 1923 | 369 |  |  |
| 1. **AD Severity (Non-atopic non-AD controls vs. Moderate-to-severe AD cases)** | | | | | |
| Dietary Pattern 3  (Meat & Rice) | Dietary Pattern 2  (Plant-based Foods) | Non-atopic Non-AD Controls | Moderate-to-severe AD Cases | SF P Value | SF Value  (95% CI) |
| Low | Low | 362 | 105 | 0.172 | 0.791  (0.565-1.108) |
| Low | Moderate-to-high | 504 | 130 |  |  |
| Moderate-to-high | Low | 861 | 277 |  |  |
| Moderate-to-high | Moderate-to-high | 1923 | 435 |  |  |
| 1. **Dietary Pattern 4 (Probiotics, Milk, & Eggs) and Dietary Pattern 2 (Plant-based Foods)** | | | | | |
| 1. **Allergic Sensitization (SPT negative subjects vs. SPT positive subjects)** | | | | | |
| Dietary Pattern 4  (Probiotics, Milk & Eggs) | Dietary Pattern 2  (Plant-based Foods) | SPT Negative | SPT Positive | SF P Value | SF Value  (95% CI) |
| Low | Low | 833 | 1656 | 0.149 | 0.884  (0.748-1.045) |
| Low | Moderate-to-high | 953 | 1727 |  |  |
| High | Low | 499 | 1106 |  |  |
| High | Moderate-to-high | 1622 | 2897 |  |  |
| 1. **Ever AD (Non-atopic non-AD controls vs. AD cases)** | | | | | |
| Dietary Pattern 4  (Probiotics, Milk & Eggs) | Dietary Pattern 2  (Plant-based Foods) | Non-atopic Non-AD Controls | AD Cases | SF P Value | SF Value  (95% CI) |
| Low | Low | 646 | 428 | 0.253 | 0.867  (0.680-1.107) |
| Low | Moderate-to-high | 772 | 429 |  |  |
| High | Low | 375 | 308 |  |  |
| High | Moderate-to-high | 1292 | 772 |  |  |
| 1. **AD Chronicity (Non-atopic non-AD controls vs. Chronic AD cases)** | | | | | |
| Dietary Pattern 4  (Probiotics, Milk & Eggs) | Dietary Pattern 2  (Plant-based Foods) | Non-atopic Non-AD Controls | Chronic AD Cases | SF P Value | SF Value  (95% CI) |
| Low | Low | 646 | 174 | 0.814 | 1.043  (0.734-1.482) |
| Low | Moderate-to-high | 772 | 134 |  |  |
| High | Low | 375 | 114 |  |  |
| High | Moderate-to-high | 1292 | 264 |  |  |
| 1. **AD Severity (Non-atopic non-AD controls vs. Moderate-to-severe AD cases)** | | | | | |
| Dietary Pattern 4  (Probiotics, Milk & Eggs) | Dietary Pattern 2  (Plant-based Foods) | Non-atopic Non-AD Controls | Moderate-to-severe AD Cases | SF P Value | SF Value  (95% CI) |
| Low | Low | 646 | 186 | 0.353 | 0.855  (0.615-1.190) |
| Low | Moderate-to-high | 772 | 182 |  |  |
| High | Low | 375 | 126 |  |  |
| High | Moderate-to-high | 1292 | 304 |  |  |

Abbreviation: Atopic dermatitis (AD), synergy factor (SF).

A SF p-value < 0.05 is statistically significant and written in bold. A SF value < 1.000 is antagonistic (counteracting interaction to reduce the overall associated odds ratios for allergic outcomes) while a SF > 1.000 is synergistic (enhancing interaction to increase the overall associated odds ratios for allergic outcomes).
